# Evaluating automated titre score as an alternative to continuous flow analysis for the prediction of passive anti‐D in pregnancy

**DOI:** 10.1111/tme.12743

**Published:** 2020-12-14

**Authors:** Michelle L. Evans, Benjamin Holmes, Kerry Dowling, Tracey Lofting, Matthew R. Barnett, Nicolette Heydon, Tracy Clarke, Christopher Hall, Eva‐Maria Surmann, Sophie C. I. Callsen, Wim Malomgre

**Affiliations:** ^1^ Blood Sciences Newcastle Upon Tyne Hospitals NHS Foundation Trust UK Newcastle upon Tyne UK; ^2^ North Lincolnshire and Goole NHS Foundation Trust North Lincolnshire UK; ^3^ University Hospital Southampton NHS Foundation Trust Southampton UK; ^4^ Southwest Pathology Services Taunton UK; ^5^ Gloucestershire NHS Foundation Trust Gloucestershire UK; ^6^ Ortho Clinical Diagnostics Raritan New Jersey USA

**Keywords:** anti‐D, automated titre, continuous flow analyser, HDFN, ortho vision, titre score

## Abstract

**Objectives:**

To evaluate the potential of the automated titre score (TS) as an alternative method to continuous flow analysis (CFA) for the prediction of the nature of anti‐D in pregnancy.

**Background:**

The 2016 revised British Society for Haematology (BSH) antenatal guidelines recommended a measurement of anti‐D concentration by CFA to ensure the detection of potential immune anti‐D. Due to high referral costs and resource pressures, uptake has been challenging for hospital laboratories. Serious Hazards of transfusion (SHOT) data have previously shown that this has contributed to missed antenatal follow ups for women with immune anti‐D and neonates affected by haemolytic disease of the fetus/newborn.

**Methods/Materials:**

In this multicentre comparative study, samples referred for CFA quantification were also tested by an ORTHO VISION automated anti‐D indirect antiglobulin test (IAT) serial dilution and then converted to TS. CFA results and history of anti‐D prophylaxis were used to categorise samples as passive or immune, with the aim of determining a potential TS cut‐off for CFA referral of at risk patients.

**Results:**

Five UK National Health Service (NHS) trusts generated a total of 196 anti‐D TS results, of which 128 were classified as passive and 68 as immune. Diagnostic testing of CFA and TS values indicated a TS cut‐off of 35 to assist in distinguishing the nature of anti‐D. Using this cut‐off, 175 (89%) results were correctly assigned into the passive or immune range, giving a specificity of 92.19% and a negative predictive value of 91.47%.

**Conclusion:**

TS in conjunction with clinical and anti‐D prophylaxis history can be used as a viable and cost‐effective alternative to CFA in a hospital laboratory setting.

## 1 INTRODUCTION

Close collaboration between the blood transfusion laboratory and obstetric teams is critical to identify the presence of maternal red cell antibodies (~1% of pregnancies)^1^ and to monitor these levels throughout the antenatal period. Historically, anti‐D was the most common cause of haemolytic disease of the fetus and newborn (HDFN), accounting for 18%–27% of all cases.^2^ The advent of anti‐D prophylaxis in the 1960s dramatically reduced the incidence of sensitisation and production of immune anti‐D. Hence, the number of deaths from anti‐D related HDFN cases reduced from 46/100000 to 1.6/100000, with a further reduction seen after the introduction of antenatal prophylaxis.^2^ The anti‐D prophylaxis programme continues to be hugely successful; however, sensitisation still occurs in ~500 pregnancies per year in the United Kingdom^3^; this would equate to ~78/100000 sensitisations to anti‐D based upon current UK live birth data.^4^

Once anti‐D has been detected in pregnancy, it needs to be quantified to categorise risk and to determine the need for further obstetric review. If the anti‐D detected is prophylactic (passive) in nature, there is no risk; however, the nature of detected anti‐D (passive or immune) cannot be determined by qualitative laboratory methods alone. The majority of transfusion laboratories in England currently refer samples to the National Health Service Blood and Transplant (NHSBT) for quantification by continuous flow analysis (CFA). The concentration of prophylactic anti‐D rarely exceeds 0.4 IU/ml^5^ by CFA, falling into the low risk of HDFN category. Difficulties in differentiating between immune and passive anti‐D can lead to prophylaxis being omitted where it is required and women not receiving appropriate follow up during pregnancy. Six cases of newborns with HDFN were reported to Serious Hazards of transfusion (SHOT) in 2012 due to this incorrect assumption,^5^, ^6^ but the CFA results and clinical impact of such cases were not provided. To address these issues, the 2016 British Society for Haematology (BSH) antenatal guidelines recommended quantification of all samples containing anti‐D by either CFA or a method that has been extensively validated against CFA.^5^ However, the referral of these samples presents hospitals with many challenges, including high referral costs and resource pressures for both the hospital and the reference lab, as well as long turnaround times for obtaining results. Due to the issues discussed, many transfusion laboratories have been unable to implement the 2016 guidelines, meaning that patients are still potentially at risk.

In a climate of austerity, hospitals must develop strategies to provide the best care in a cost‐effective manner. Some blood transfusion automated systems have the ability to perform automated titrations. Currently, reporting this method as an endpoint titre is considered to be semi‐quantitative, does not accurately represent the clinical picture and correlates poorly with the severity prediction of HDFN.^7^ The adoption of a titre score (TS), however, provides a more quantitative result that takes into account the strength of the reaction and the avidity of the antibody and is thought to better correlate with risk.^7^, ^8^, ^9^ The aim of the first phase of this study was to assess if TS determined by automated ORTHO BioVue column agglutination technology (CAT) is a comparable alternative to the existing CFA for the categorisation of the nature of anti‐D (prophylactic or immune).

## 2 MATERIALS AND METHODS

Samples from pregnant women collected between April 2017 and December 2018, who were found to have a detectable anti‐D (passive or immune), were included in the study.

A total of 196 samples were tested across five UK hospital transfusion laboratories using 10 ORTHO VISION platforms. All laboratories participating in the study referred whole‐blood expaned to ethylenediamine tetraacetic acid samples from pregnant women with detectable anti‐D to the NHSBT red cell immunohaematology (RCI) laboratory for anti‐D quantification by CFA. Prior to referral, these samples were tested on the ORTHO VISION platform, an automated TS was determined and the results were compared with the CFA result.

Ethical approval was not obtained as only the CFA result was reported to the clinician; no additional samples were requested; and only samples with enough volume were tested in house prior to referral, thus not impacting patient care or management.

Samples with detectable anti‐D were quantified at RCI laboratories using Astoria 2 flow analysers employing the reference method (White Horse Scientific Ltd. Pewsey, Wiltshire, United Kingdom).

**FIGURE 1:**
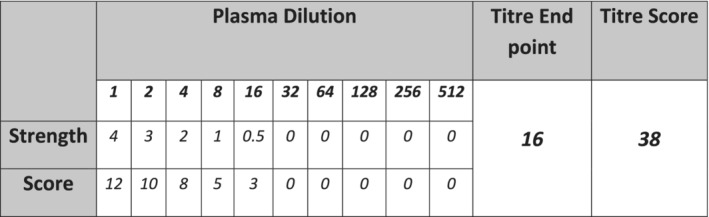
A worked example of titre score (TS) application

The ORTHO VISION platform automated serial dilution functionality was used to make doubling dilutions of the patient's plasma in standard phosphate‐buffered saline solution. Serial dilutions were prepared from neat to 1in 1024. The assay included a negative control to test for any antibody carryover from the neat plasma. These dilutions were tested against a pooled OR1r 0.8% reagent red cell (ref NHSBT PR045) using BioVue Anti‐IgG cassettes (Ortho Clinical Diagnostics ref 707 450). The reaction grades were then read by the ORTHO VISION Cassette Imaging System (CIMS) and reported by the Image Processing System (IPS). Each positive reaction grade was manually converted into a score value (Table 1), and the sum of all scores gave the TS for each individual sample. See a calculated TS example in Figure 1.

**TABLE 1:** Automated titre reaction strengths are converted to titre score (TS) value

IAT reaction strength	4	3	2	1	0.5	0
Score value	12	10	8	5	3	0

The NIBSC anti‐D standard (NIBSC Ref 73/515) was used as a quantitative positive control with a known concentration of 0.23 IU/ml.

To aid statistical analysis and result interpretation, the nature of the anti‐D was assigned. Anti‐D was classified as passive based on a combination of a CFA result of less than 0.4 IU/ml, patient clinical history and applicable evidence of anti‐D prophylaxis and D status of the child where available. If no evidence to suggest a passive nature was identified, the antibody was assumed to be immune.

## 3 DATA ANALYSIS

The CFA quantification results from NHSBT were directly compared to the TS. Diagnostic test (2 × 2) analysis was performed using MedCalc statistical software (© 2020 MedCalc Software bv). Sensitivity, specificity and positive/negative predictive values (NPVs) were determined at various TS cut‐off points and used to propose the most appropriate score to differentiate between passive and immune anti‐D in conjunction with patient clinical history.

## 4 RESULTS

**FIGURE 2:**
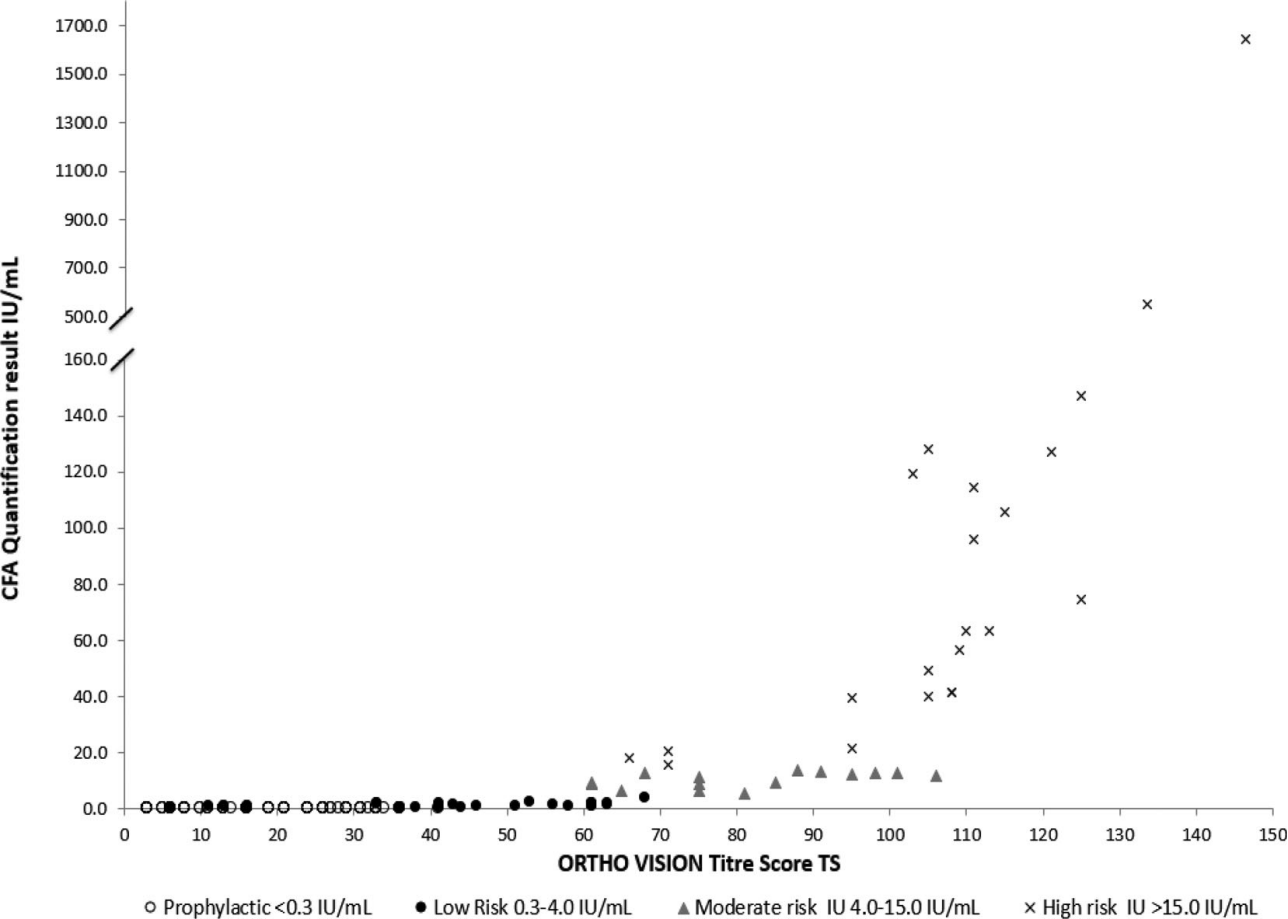
Titre score (TS) versus continuous flow analysis (CFA) quantification based upon haemolytic disease of the fetus/newborn (HDFN) risk categories

**FIGURE 3:**
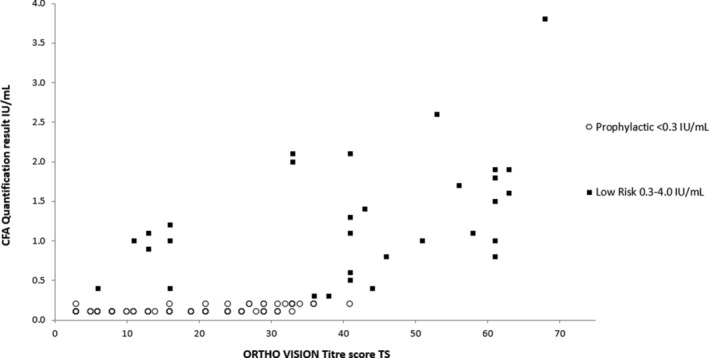
Passive and low‐risk titre score (TS) versus continuous flow analysis (CFA) comparison

A total of 196 samples were tested across five UK hospital transfusion laboratories using 10 ORTHO VISION platforms. Of the anti‐D detected in these samples, 128 were classified as passive and 68 as immune. The TS and CFA values were compared and presented based upon the HDFN clinical risk categorisation (prophylactic/low/moderate/high) (Figure 2).^5^, ^10^ The suggestive TS cut‐off value range between prophylactic and the low‐risk immune category results was informed by Figure 3. Diagnostic testing was then performed to define potential TS values (Table 2).

**TABLE 2:** Diagnostic testing of potential titre score (TS) cut‐off values

Titre score	30	31	32	33	34	35
True positives (TP)	59	59	59	57	57	57
True negatives (TN)	106	110	111	117	118	118
False positives (FP)	22	18	17	11	10	10
False negatives (FN)	9	9	9	11	11	11
Sensitivity (%)	86.76	86.76	86.76	83.82	83.82	83.82
Specificity (%)	82.81	85.94	86.72	91.41	92.19	92.19
Positive predictive value (PPV) (%)	72.84	76.62	77.63	83.82	85.07	85.07
Negative predictive value (NPV) (%)	92.17	92.44	92.50	91.41	91.47	91.47

*Note:* TP All patient titre scores > 35 assumed immune, FP All patient titre scores > 35 assumed prophylactic, TN All patient titre scores ≤ 35 assumed prophylactic, FN All patient titre scores ≤ 35 assumed immune.

The mean quantification value was 22 IU/ml with a median of 0.2 IU/ml (range < 0.1–1643.6 IU/ml). The mean and the median TS values were 35 and 24, respectively (range 3–148).

Potential TS cut‐off values were selected for analysis based on a passive and immune low‐risk boundary as identified in Figures 2 and 3.

Using a TS cut‐off value of 35, 175 (TP + TN)/196 (89%) were correctly classified as passive or immune. A TS cut‐off value of 35 correctly categorised 118 (TN)/128 (92%) samples confirmed as prophylactic anti‐D, signifying an NPV of 91.47% (95% CI of 86.17%–94.86%) and a negative likelihood ratio of 0.18.

For the detection of immune anti‐D, a TS cut‐off value of 35 correctly categorised 57 of 68 (83%) confirmed as immune anti‐D by CFA, signifying a positive predictive value of 85.07% (95% CI of 75.70%–91.25%) and a positive likelihood ratio of 10.73.

## 5 DISCUSSION

The recommendation in the 2016 BSH antenatal guidelines^5^ for all anti‐D detected in pregnancy to be quantified by CFA has a potential financial impact on hospital transfusion laboratories due to increased referral and turnaround times. The findings of this study demonstrate that an automated TS determined using ORTHO VISION can reliably predict the nature of anti‐D detected in pregnancy as passive with a sensitivity of 83% and specificity of 92% when using a TS of 35.

CFA is recognised in the United Kingdom as the preferred technique for anti‐D quantification^2^ with cut off values embedded in clinical follow‐up criteria.^5^, ^10^ CFA has already been previously compared to CAT titration values using a TS method,^8^ which was described as showing promising comparability and intra‐laboratory reproducibility. This study supports such findings by demonstrating parity between the two methods in determining passive and immune anti‐D (Figure 2). Generally, where a high quantification result was reported, a high TS was also observed. Similarly, for low quantification, a low TS was observed. From reviewing Figure 1, we were unable to demonstrate a linear correlation between the TS and the concentration of anti‐D in IU/ml. However, there appear to be emerging risk groups that will be further analysed as part of an ongoing study. Such comparable clinical decision and risk correlations have already been observed between CFA anti‐D concentrations and anti‐D titres in other studies.^11^

Manual titration methods, both tube and column agglutination indirect antiglobulin test (IAT), have been associated with inter‐laboratory variation due to the preparation of the reagents and serial dilutions, as well as the visual interpretation of the end result.^12^ However, ORTHO BioVue Column Agglutination has been shown to be an appropriate replacement for tube IAT in antibody titration^13^ by reducing some of the variability factors. Utilising an automated analyser to perform titration serial dilutions further improves standardisation and turnaround time and reduces the risk of errors associated with manual testing and interpretation.^14^ Internationally, a titre endpoint may be used routinely as a clinically actionable result; however, it has also been widely critiqued as poorly correlating with regard to the severity of HDFN.^8^ Titre endpoint is thought to be a semi‐quantitative estimate and does not evaluate the strength of the reaction obtained,^8^ whereas TS has been shown to be sensitive to a wide range of antibody levels as determined by CFA and takes into account analytical variation.^7^, ^8^

Methodology on its own cannot be used to determine the nature of anti‐D but should be used in conjunction with confirmed clinical history that includes evidence of anti‐D prophylaxis date and dose.^3^ With the use of a TS of >35 as a cut‐off for CFA referral, 10 of 196 (5.1%) results were false positives. These samples would have been referred when they were in fact prophylactic in nature. In routine clinical practice, these samples would still have been referred under the current BSH guidelines, representing no clinical impact.

In addition, 11 of 196 (5.6%) samples were false negatives. These samples would not have been referred based on the TS alone. When taking into account patient clinical history, none of these patients had evidence of anti‐D prophylaxis and therefore would all have been referred for CFA for the duration of the pregnancy, all being classified as immune in nature. Looking in detail at these 11 samples, 6 (3% of 196) were taken from one patient who consistently showed a low TS (6–16) with a low‐risk category CFA result (0.4–1.2 IU/ml) throughout the duration of the pregnancy. In addition, four (2% of 196) would also have been predicted to be passive in nature based on CFA result. One patient was early in gestation with known anti‐D and anti‐G. In the absence of evidence of prophylaxis, all 11 samples would have been referred for CFA regardless, and as such, there is no clinical risk associated with this finding.

A decision‐making algorithm should accompany any implementation of this method to aid interpretation and clinical decision‐making. An example algorithm has been included in Appendix 1.

## 6 CONCLUSION

In this study, the ORTHO VISION fully automated platform has provided the ability to standardise the TS methodology across multiple sites and systems, thereby removing variability inherent to manual titration techniques.

Using a TS cut‐off value of >35, there was no additional clinical risk when facilitating conformity to BSH guidelines, thereby reducing the number of samples referred for CFA quantification and supporting the prevention of incidents related to the mis‐categorisation of the nature of anti‐D, as noted in the 2012 SHOT report.^6^

Some hospitals involved in this study are currently in the process of implementing this method, which will be used to support decision‐making algorithms for appropriate referral for CFA. This could be beneficial to the obstetric department by reducing turnaround times, offering potential financial savings and improving patient care.

The next phase of the study will involve gathering further data on women with immune anti‐D, correlating the automated TS with CFA and clinical risk categories and outcomes. This will include inter‐laboratory variation studies.

*Disclaimer*: The data and conclusions above are specific for the ORTHO VISION platform and ORTHO VISION technology. Other methodologies may not yield equivalent results and should be validated appropriately.

## AUTHOR CONTRIBUTIONS

**Michelle L. Evans, Benjamin Holmes, Kerry Dowling, Tracey Lofting, Matthew R. Barnett, Nicolette Heydon** and **Tracy Clarke:** Performed the research. **Michelle L. Evans, Benjamin Holmes, Kerry Dowling, Tracey Lofting, Matthew R. Barnett, Nicolette Heydon, Tracy Clarke, Christopher Hall, Sophie C. I. Callsen**, and **Wim Malomgre:** Designed the study. **Michelle L. Evans, Benjamin Holmes, Kerry Dowling, Tracey Lofting, Matthew R. Barnett, Nicolette Heydon, Tracy Clarke, Christopher Hall, Sophie C. I. Callsen, Wim Malomgre** and **Eva‐Maria Surmann:** Analysed the data, wrote and revised the paper. **Christopher Hall, Sophie C. I. Callsen, Wim Malomgre** and **Eva‐Maria Surmann:** Contributed essential reagents and tools.

## CONFLICT OF INTERESTS

The authors have no competing interests.
